# Relationship between Dough Properties and Baking Performance of Panned Bread: The Function of Maltodextrins and Natural Gums

**DOI:** 10.3390/molecules28010001

**Published:** 2022-12-20

**Authors:** Abdulmajeed A. AbuDujayn, Abdellatif A. Mohamed, Mohamed Saleh Alamri, Shahzad Hussain, Mohamed A. Ibraheem, Akram A. Abdo Qasem, Ghalia Shamlan, Nashi K. Alqahtani

**Affiliations:** 1 Department of Food Science and Nutrition, King Saud University, Riyadh 11456, Saudi Arabia; 2 Department of Food and Nutrition Sciences, College of Agricultural and Food Sciences, King Faisal University, Al-Ahsa 31982, Saudi Arabia

**Keywords:** maltodextrins, ziziphus, bread, sensory, dough

## Abstract

The effectiveness of hydrocolloids (2% maximum in various combinations) from various sources, including maltodextrins (MD) with polymerization degree (DP) 18 and ziziphus gum (ZG), on the dough properties and quality of panned bread, as well as the possibility of using them to delay the bread staling process, have been investigated after 24, 72, and 96 h of storage. By evaluating the pasting capabilities of wheat flour slurry, dough properties, and the final product, the effects of the ziziphus gum (ZG) and maltodextrins (MD) were ascertained. A TA-TXT texture analyzer, a texture profile analysis test, and Micro-doughLab were used to evaluate the dough mixing properties. Additionally, a hedonic sensory evaluation of the overall acceptance of the bread, as well as its texture, aroma, taste, and color, was done. It is clear that MD had a more distinct impact than ZG on the way dough was mixed, the texture of the gel, and the finished product. The combination of MD and ZG significantly altered the bread’s physical characteristics and its aging over time. The decreased peak viscosity and noticeably smaller setback of wheat flour gels, which corresponded to lower gel hardness, serve as evidence of reduced amylose retrogradation. At 2%, MD outperformed ZG in terms of increasing water absorption, dough stability, and bread loaf volume. With the exception of the blend that included three times as much MD as ZG, all mixes, including the control, exhibited an increase in bread firmness as a function of time after storage. Overall, the panelists liked (score of 5 and above) the bread made with mixes that had either MD or ZG, or a combination of both.

## 1. Introduction

In recent years, the usage of additives in baked products has increased significantly. Their use aims to enhance the qualities of dough handling and the quality of freshly baked bread, and extend the shelf life of stored bread. Numerous additives with different chemical structures are employed with this goal in mind. A family of food additives known as hydrocolloids is extensively employed in the food industry, including baking. Hydrocolloids are used in the baking industry to enhance bread qualities, dough performance and the overall sensory attributes [1]. They have also been demonstrated to stop crumb texture from altering while being stored (antistaling effect). Gums, a group of hydrocolloid chemicals, are used to stabilize foams, emulsions, and suspensions, which can change the rheology and texture of aqueous systems [2]. They have been demonstrated to be powerful antistaling agents, particularly when used in cutting-edge technologies. These additives can be used to make up for the damage caused by dough freezing [3]. The dough’s water absorption increased by 1.6% after the addition of 0.2% xanthan gum, while the mixing tolerance index and degree of softening were reduced by 40 Brabender Units (BU). When applied at a 0.2% level or less, gum had no impact on the starch’s pasting peak or the gelatinization temperature. Hydrocolloids are expected to increase water retention and loaf volume, while decreasing stiffness and starch retrogradation when used in small amounts (1%) [1,4]. The extremely hydrophilic nature of hydrocolloids also helps to minimize the growth of ice crystals and water migration during frozen storage, which enhances the dough’s freeze/thaw stability. Due to its hydrophilic structure, which enables it to interact with water and boost the interface activity between water and the non-aqueous phases of the bread dough. Hydroxypropylmethylcellulose (HPMC) has the ability to function as a bread improver, which promotes the production of emulsions and sturdy uniform films [5]. Due to their great capacity for retaining water, some hydrocolloids, such as locus bean gum, are reported to have a softening impact on baked goods. Xanthan gum and alginate, on the other hand, are said to prevent gluten–starch interactions. The rheological characteristics of doughs made with wheat flour were modified to diverse degrees by hydrocolloids with different chemical structures (xanthan, sodium alginate and a *k*-carrageenan) [6]. The biggest effects of these gums were seen at the dough level, because xanthan gum was shown to strengthen the network of wheat flour dough. Additionally, fermentation tests show that xanthan is suitable for use as an ingredient when lengthy fermentation procedures are desired [7,8]. Although it is clear from the preceding that hydrocolloids are widely used in the production of bread, the quality of hydrocolloids varies substantially depending on their place of origin and chemical structure [9].

The tree species *Ziziphus Spina-Christi* is a member of the Rhamnaceae genus of plants. From Mauritania to the Red Sea, it grows throughout a considerable portion of Africa. According to the literature, the ethanol extract from ziziphus fruits possesses rheological qualities superior to guar gum and similar to xanthan gum [10]. Ziziphus mucilage should be extracted using 1:7 water at 60 °C, and then precipitated using 1:3 ethanol. The water and oil holding capacities were found to be 73.35 g per gram and 4.97 g per gram of the dry sample, respectively [11,12]. The fruits of the majority of ziziphus species vary in size, shape, color, and weight [13]. According to the reported statistics, gum ziziphus has a greater capacity for oil absorption than guar or xanthan gum, but a lower capacity for emulsification. The five phenolic compounds discovered in the extract of ziziphus are phlorizin, catechin, gallic acid, chlorogenic acid and caffeic acid [14]. Ziziphus gum was reported in the literature to modify starch pasting properties, improve wheat flour dough properties and enhance the baking qualities of breads and pastries [15,16].

Maltodextrins are non-hygroscopic, have strong water-holding properties, and are cold water-soluble. They also have a bland taste that is mild or non-sweet. They serve as bulking agents, transporters for artificial sweeteners, taste enhancers, flavor and seasoning spray-drying aids, and fat substitutes. Commercial maltodextrins come from three botanical sources: rice, potato, and corn starches [17]. Maltodextrins from various botanical sources may display varied properties because of their various parent starches, and due to inherent differences in their chemical structures [18]. Previously, researchers showed that when the amount of potato maltodextrins is increased, the falling number and water absorption of triticale flour decreased. The dough was more stable as the maltodextrin content increased, the dextrose equivalent (DE) values increased, and the dough’s stability improved, while its resistance to mixing decreased [19]. Malto-oligosaccharides with polymerization degrees (DP) of 2 to 5 inhibit the formation of amylose helices, reducing the degree of retrogradation of wheat starch, while those with a DP greater than 6 may form by themselves small helices that co-crystallize with starch polymers, accelerating retrogradation, according to Smits, et al. [20]. Wang and Jane [18] discovered that the level of starch retrogradation increased at 20 °C in the presence of high-molecular-weight malto-oligosaccharides. Commercial enzymes that were used as antistaling agents for bread in commercial settings decreased amylopectin retrogradation. This revealed a connection between bread staling and amylopectin retrogradation, and that the used maltodextrins are promising antistaling ingredients. Additionally, the findings imply that the antistaling action brought on by antistaling enzymes may very well be caused by starch hydrolysis byproducts of enzymatic attack [21]. α-amylase was used to reduce starch retrogradation. The findings show that differential scanning calorimeter (DSC) provides a measurement of both overall order and recrystallized amylopectin (recrystallized amylopectin and double-helical content). The reduced rates of retrogradation did not appear to be caused by the malto-oligosaccharides that the enzymes created, but rather by the degree of starch modification that was responsible for inhibiting retrogradation [22]. The main objective of this study was to explore the impact of maltodextrins and ziziphus gum on the dough properties and quality attributes of pan bread.

## 2. Results and Discussion

### 2.1. Pasting Properties

Table 1 lists the mixes’ and the control flour’s pasting properties. The blend’s peak viscosity (PV) was decreased by roughly 7% with respect to the control. If we assume that starch is the main contributor to PV and that this is a replacement rather than an addition, the reduction in PV should be roughly 2%. Therefore, in addition to the absence of the starch, the inclusion of maltodextrins (MD) and ziziphus gum (ZG) further decreased the PV. Both MD and ZG at 2% significantly decreased the PV (*p* ≤ 0.05); however, MD at 2% had a more pronounced effect than ZG [23]. When 2% of MD was added to flour suspension, the largest loss in PV was from 2964 to 2522 cP, while the maximum loss was from 2694 to 2609 cP when 2% ZG was added (Table 1). Regardless of their ratio, MD and ZG together also decreased the PV. Therefore, neither MD nor ZG were essentially more influential on the synergy created by their combination. PV may have decreased as a result of either a significant decrease in the starch’s ability to swell when heated or an acceleration of swelling that causes faster gelatinization. According to the findings in the literature, some gums increase PV while others suppress it. Gums such as xanthan, guar, locust bean, alginate, and cordia promoted granule swelling and raised the PV, but okra gum was found to lower the PV of several starches by inhibiting starch swelling [9,24]. Gum interactions with leached amylose molecules during the initial stages of starch gelatinization have been linked to increased viscosity during starch pasting, which may help to explain the positive impacts of hydrocolloid on PV. According to the aforementioned results, the MD and ZG presence led to changes in the wheat flour paste’s maximum PV, which were mostly brought on by interactions between the MD and ZG and the starch granules. Maximum PV reflects the starch granules’ capacity to swell freely prior to physical breakdown. The data in Table 1 show that MD and ZG decreased starch granule swelling, whereas 2% MD or ZG, as well as their combination, inhibited it, resulting in lower PV for the flour suspension. When compared to pure starch, other ingredients in the flour change the starch’s ability to paste, despite the fact that starch is the most crucial component of wheat flour in terms of pasting qualities. As a result, as other researchers have noted, there may be some interaction between the hydroxyl groups of the starch and the gum, but it is only restricted in the flour solution [25]. The hydrocolloids, according to some researchers, are only present in the continuous phase of the medium, and as long as the starch granules swell, the concentration of the hydrocolloids within the continuous phase increase, causing a significant increase in the continuous phase’s viscosity. Other researchers view starch pastes as suspensions of swollen granules dispersed in a continuous macromolecular medium [26].

However, the setback revealed no significant differences between the control and the mixtures, while the breakdown and final viscosity of the wheat suspension followed the same trend as the PV (Table 1). The breakdown of viscosity reveals how much viscosity is lost. Higher breakdown was brought on by more ZG. Although the difference was not significant, MD and ZG in equal amounts brought on the least breakdown. This outcome is consistent with other studies in which certain hydrocolloids promoted breakdown while others inhibited it, which may have been caused by the molecular structure of the gums [27]. As the breakdown values rise, the starch granules’ resistance to thermal treatment and mechanical shear decreases. Setback is the rise in viscosity that occurs when amylose retrogrades during the cooling process after starch gelatinization. The blend with 2% ZG led to a higher setback, whereas the sample with equal levels of MD and ZG had the least. Low setback suggests amylose and added ingredients interaction, which is illustrated by the addition of an equal amount of MD and ZG to the flour suspension. Given that MD has been demonstrated to lessen amylose retrogradation and shear stress, a reduction in setback caused by MD is expected [28].

At 2% concentrations, maltodextrins significantly (*p* ≤ 0.05) raised the pasting temperature (PT) of the flour suspension, but ZG and the combinations had non-significant effects irrespective of the mixture’s composition. MD and ZG had no concentration- or ratio-dependent effects. This information is consistent with reports in the literature [9]. The PT was reported to be decreased even further at the 1% concentration of xanthan gum. This tendency was seen for gums other than the ones tested here. The PT decrease is critical because it signifies the earlier beginning of starch gelatinization. This means that there will be more starch accessible as an enzyme substrate during baking, which will reduce the loaf volume of the bread.

### 2.2. Textural Properties of Flour Mixes

The data in Table 2 demonstrate that MD and ZG significantly (*p* ≤ 0.05) decreased gel hardness, regardless of which agent was used alone or in conjunction with the other. Gel hardness readings varied from 77.3 g to 67.3 g, whereas the control showed an average of 85.0 g. The mixture with 1.5% MD and 0.5% ZG had the lowest hardness measurement, whereas the sample with 2% MD in comparison to the control had the greatest. As seen for BF2 and BF3 in Table 2, a higher ZG concentration was more effective in lowering hardness. The hardness value was lowest among all combinations, when 1.5% MD and 0.5% ZG were combined. Starch gel retrogradation, which is regulated by amylose and amylopectin chain rearrangement, is what mostly causes hardness. This suggests that the decrease in hardness may be caused by MD or ZG interfering with the formation of ordered structures during starch retrogradation by forming hydrogen bonds with amylose molecules [29]. Since gel setback and gel hardness are outcomes of amylose retrogradation, it is anticipated that MD and ZG will have a similar impact on these parameters. In actuality, the sample with 1.5% MD and 0.5% ZG recorded the lowest gel hardness and 2% MD the highest compared to the control, whereas the sample with 1% MD and 1% ZG recorded the least setback and a sample with 2% ZG gave the highest setback. As a result, the sample with the least setback is generally regarded as the best. This discrepancy is a result of the measurement conditions, as setback was recorded at 50 °C, while hardness was evaluated at room temperature.

The amount of energy required to overcome the forces of attraction between the surface of the food and the surfaces of other materials it comes into direct touch with is known as adhesiveness. Measurements of adhesiveness ranged from 0.73 mJ (control) to 1.07 mJ (BF2). Samples with MD and ZG required greater force to penetrate the gel’s adhesiveness. Even though the control and mixed samples did not differ significantly from each other, those with 2% ZG required more energy. Similar to the setback, the 2% ZG mix once more exhibited extreme results. The promotion of starch–starch interaction by MD and ZG could explain the increase in gel adhesiveness. Cohesiveness is the measure of how much a material can be deformed by the applied force before breaking. Cohesiveness significantly (*p* ≤ 0.05) decreased with the addition of MD or ZG, with the sequence being control > BF1; BF4 > BF5; BF2; BF3. This demonstrates that the MD-containing gel and the control gel are more resistant to degradation before it happens. Gumminess, which is the result of hardness times by cohesiveness, directly reflects the energy needed to break down food before swallowing. Given that gumminess depends on hardness and that MD and ZG significantly reduced gumminess, the control showed significantly greater gumminess and hardness even if the adhesiveness of the control and the mixes was similar. According to Yildiz, et al. [30], the addition of apple fiber reduced the gumminess, chewiness, and hardness of wheat starch gel. Despite the fact that the wheat flour tested here contains components other than just wheat starch, these results are consistent with them. None of the gel parameters assessed in this study were concentration-dependent (up to 2%), meaning that adding more or less MD had no significant impact on these variables.

### 2.3. Dough Mixing Properties

The dough mixing quality was determined using DoughLab, as shown in Table 3. Water absorption (WA) is the quantity of water required to attain 500 BU in 2 min after mixing begins. This study shows that the addition of MD or ZG greatly increased the quantity of water required for dough development, but that the influence on water absorption was greater when MD or ZG were introduced separately. The WA value fluctuates according to the chemical make-up of the added components and their capacity to absorb water; a high WA of additional ingredients will obstruct flour’s ability to absorb water and form the desired dough. Other gums such as guar gum and cordia gum were reported to decrease WA [3]. The term “dough development time” (DDT) describes the period of time needed after mixing flour dough for it to reach its maximum consistency. The DDT ranged from 1.7 to 6.43 min, with the control requiring the lowest DDT and the blend with 2% MD requiring the most, while the other mixes were remarkably similar. Although it disagrees with the effects of k-carrageenan or HPMC, this study supports claims that adding hydrocolloids such as xanthan, alginate, or guar increased DDT [7,14]. The dough matrix takes longer to form when hydrocolloids are added, raising the DDT, and vice versa when the DDT is decreased. The competition for water or the interaction of the developing gluten with MD or ZG could both contribute to the delay. The ability of the dough to keep its consistency over time indicates its mechanical strength, measured by its dough stability. Both 2% MD and 1.5% MD+ 0.5% ZG markedly improved dough stability; nevertheless, the 2% ZG had the shortest stability time i.e., 5.90 min as opposed to 5.93 min for the control. Since all of the samples that contained MD had high dough stability, it may be inferred that MD had a greater effect on dough stability, whereas ZG had a negative effect on stability (Table 3). These data are consistent with studies in the literature finding that various gums reduce dough stability [7]. MTI, which represents dough softening during mixing, is the difference between the BU at the peak of the curve and the value at the peak of the curve five minutes later. An MTI value of 30 B.U. or fewer is regarded as exceptionally good for bread wheat flours because it indicates how well the flour mixes. A flour with an MTI more than 50 FU has a reduced tolerance for mixing and is more likely to lead to issues when handled mechanically, as well as in dough formation. Regardless of the quantity, samples containing ZG significantly raised the MTI of the control, whereas no significant change was observed when MD was added to the blend. In contrast to ZG, which indicates dough instability during mixing, this tendency is similar to the effect of MD on dough stability, indicating stability and higher machinability. Strong gluten hard red spring wheat flour’s MTI was observed to be lowered than other gums such as xanthan, guar, and alginate at 2% and higher, whereas alginate at 2% reduced MTI to zero FU [31]. FQN is the distance in mm along the time axis from the point of water addition to the point where the height at the center of the curve has decreased by 20 BU. More ZG-containing samples considerably (*p* ≤ 0.05) reduced FQN, but more MD-containing samples significantly *(p* ≤ 0.05) increased FQN. The MTI trend and FQN trend are comparable. The reduction in FQN could be attributed to gluten–ZG gum interaction. The FQN of wheat flour has been shown to be reduced by rice bran and bagasse fiber as a result of the interaction between gluten and fiber [32]. The FQN results show that wheat flour mixes with 2% and 1.5% MD are still appropriate for bread baking. The degree of dough softening can be determined by comparing the consistency value of the dough mixing curve center at the end of the developing time with the curve center 12 min later. Dough softening was increased by ZG and ZG/MD-mixes, but not by MD alone. Softening can be ordered in the following manner: BF4; BF3 > BF2; BF5 > control > BF1.

### 2.4. Wheat Flour Dough Extensibility

The strength of the dough is its extensibility, which also contributes to the final baked good’s consistency and quality. Knowing what extensibility is, though, is not sufficient. In dough, extensibility is one part of a delicate balancing act that starts with mixing. Both extensible and elastic characteristics, and the capacity to stretch and regain the original shape, are developed when the gluten matrix is formed. When the dough expands and contracts during the fermenting, proofing, or baking process, it will not perform properly if one of these characteristics is stronger than the other. The dough extensibility and resistance to extension are presented in Table 4. The greatest peak force used here to assess dough extensibility serves as a measure for the sample’s tensile strength (also known as the elastic limit): the higher the value, the more elastic the dough is. Once the dough has reached its elastic limit, deformation starts to take place and eventually breaks. Since both dough strength and dough extensibility are desired, a negative relationship between them would be problematic for developing hard wheat varieties for bread making. While BF2, BF3, and BF5 were the least resistant to extension compared to the control, BF1 (2% MD) was the most. Once more, ZG had a worse impact on the control dough than MD. This pattern matched the impact of ZG on dough stability, as displayed in Table 4. The dough network is a continuous phase represented by high-molecular-weight glutenin, which is contributed by the development of disulfide bonds and is portrayed by the elastic property, and a discontinuous phase symbolized by the low-molecular-weight gliadin that is indicated by the viscous property. Due to their lower molecular weight than glutenin, MD and ZG are located in the discontinuous phase, which helps to maintain a balance between the extensibility and elasticity of the dough. This fact is clear from Table 4’s extensibility data, which show that both MD and ZG significantly (*p* ≤ 0.05) increased dough extensibility in the order of BF4; BF1; BF3 > BF2 > control. Some gums, such as xanthan gum, have been found to lessen the extensibility of dough. All mixes will perform well for baking bread, and based on the extensibility test, the mixes with 2% MD and equal parts MD and ZG will perform particularly well.

### 2.5. Solvent Retention Capacity (SRC)

SRC testing was initially established to assess the functionality of soft wheat flour. Damaged starch, gluten proteins, and arabinoxylans make up the majority of the functional ingredients in flour. The SRC test is a solvation assay for flours that determines the improved swelling behavior of certain polymer networks in a limited number of diagnostic solvents to estimate the functional contribution of each distinct flour component. Throughout, the water retention capacity (WRC) of flour is influenced by all water-absorbing ingredients. Lactic acid retention capacity (LaRC) is connected to the characteristics of the gluten protein, sodium carbonate retention capacity (SCRC) is related to the amount of damaged starch, and sucrose retention capacity (SuRC) is linked to pentosans. Regardless of composition, adding MD and ZG caused a small rise in the WRC of the control flour with no significant difference between the mixes (Table 5). Other gums such as cordia gum increased the WRC of wheat flour [16]. WRC varied from 76.07 (control) and 92.83 (1% + 1%). While high ZG concentrations (2%) maintained the same SuRC as the control, the SuRC data reveal a considerable reduction with greater MD content, with the 2% MD exhibiting the lowest SuRC. Gaines [33] noted a significant rise in SuRC as a result of the gum addition. The type of flour used and the chemical make-up of the gum added may be the causes of this discrepancy between the data presented here and in the literature reports. The 2% MD, 1.5% MD + 0.5% ZG combination significantly increased the SCRC, which is related to starch degradation of the flour, but the other mixtures were comparable to the control. The behavior patterns of the SCRC values are associated with the performance of flour in baking across a range of end-use applications, including bread baking. Because starch damage is a major factor that influences flour quality, strong gluten flour (bread flour) is expected to have high LARC, but soft wheat flour has low LARC, according to reports in the literature.

Soft wheats with relatively high LARC values have strong gluten and work best for flat bread, whereas those with low LARC values have weaker gluten and work best for pastries, according to Guttieri, et al. [34]. The control in Table 5 has a high LARC, which indicates that it is ideal for baking bread. However, the addition of MD and ZG increases LARC, making the mixture even more suitable, as demonstrated by the order of the LARC values: BF4 > BF3; BF5 > control, BF1, BF2.

### 2.6. Bread Color, Volume and Firmness Evaluation

**Figure 1:**
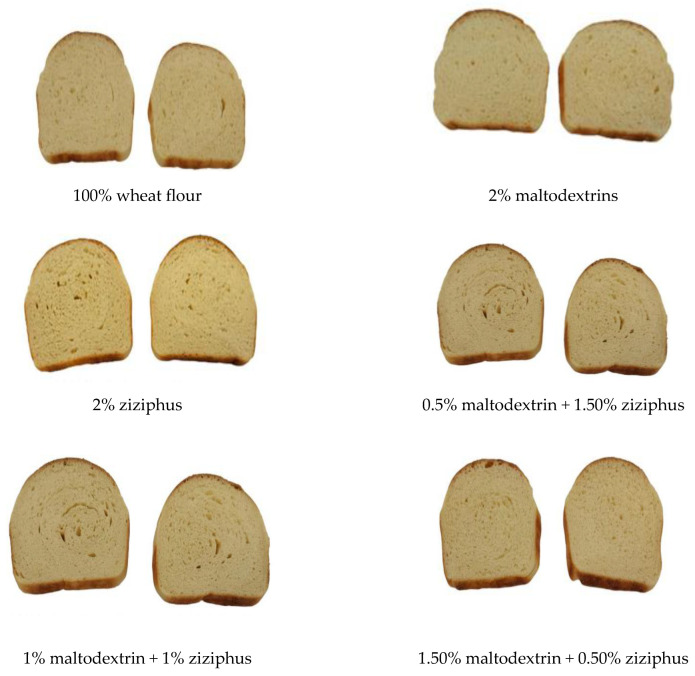
Pictures of bread slices highlighting their crumb grain and structure.

**Figure 2:**
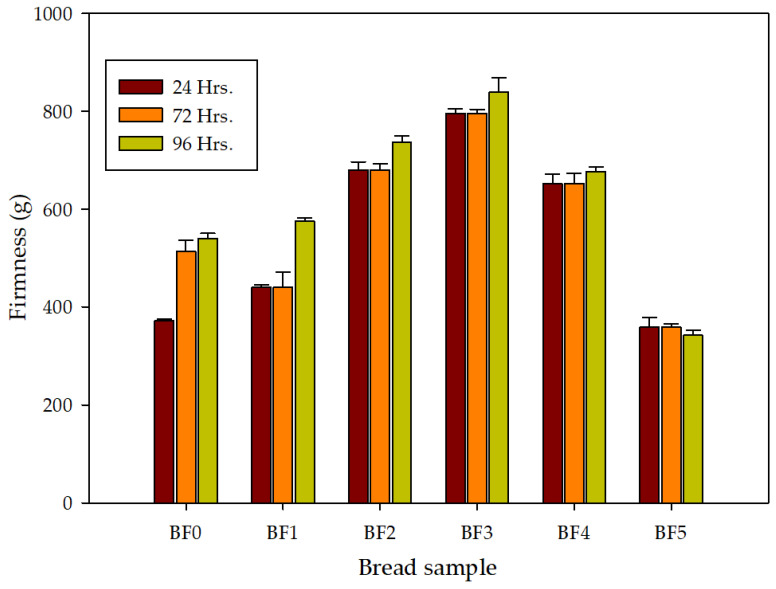
Effects of maltodextrin and ziziphus gums on the firmness of bread samples. BF: control (100% wheat flour). BF1: 98% wheat flour + 2% maltodextrin. BF2: 98% wheat flour + 2% ziziphus gum. BF3: 98% wheat flour + 0.5% maltodextrin + 1.5% ziziphus gum. BF4: 98% wheat flour + 1% maltodextrin + 1% ziziphus gum. BF5: 98% wheat flour + 1.5% maltodextrin + 0.5% ziziphus gum.

The data in Table 6 demonstrate how the inclusion of MD and ZG considerably (*p* ≤ 0.05) decreased the loaf volume of the control. This contradicts other findings in the literature, in which the authors claimed that adding guar and xanthan gums and a cellulose derivative increased the loaf volume [35]. Because the water molecules connected to the side chains of the gums are released when the hydrated chains of these gums are heated to high temperatures, there is a tighter connection between the chains in the case where gums enhance the loaf volume of the bread. These chains connections result in the formation of a temporary network. This could be the reason why these gums increase bread volume. This network will assist the dough’s gas cells during the initial baking process. As the bread bakes, the dough’s gas cells expand, reducing gas losses and increasing loaf volume. Although we observe an increase in water absorption as a result of MD and ZG, it appears that the water is tightly bound to MD and ZG, which restricts the creation of chain–chain networks and inhibits the ability of the dough network to support itself and reduce gas loss during baking [35,36]. Despite the difference in the quantity of water absorption observed during dough mixing, bread samples with higher MD demonstrated higher loaf weight compared to the control and ZG-containing bread (Table 6), indicating the potential of MD to hold water (Table 3). Crumb color showed significant alterations (*p* ≤ 0.05) in bread with MD and ZG. When compared to the control, the presence of MD made the crumb lighter (L*), whereas the ZG made it darker than the control. The sample with 2% MD had the greatest L* value, whereas the sample with 2% ZG was darker (Table 7). When examining samples with MD, it is clear that the red (a*) and yellow (b*) colors seemed to grow with an increase in ZG. The images in Figure 1 demonstrate how the grain of the slices—their porosity—reflects the firmness of the bread, with the high porosity of sample BF4 (1% MD and 1% ZG) reflected in the lighter color (high L*), and the firmer bread resulting from sample BF3 (0.5% MD + 1.5 ZG). After 24 h, the firmness of the bread ranged from 760 to 352, with the sample with the greatest ZG content (1.5%) and the other sample having an equal quantity of MD and ZG (Figure 2). Higher ZG created stiffer bread after 72 h, but higher MD produced a softer bread crumb. The same trend continued after 96 h of storage. In the findings reported here, the hardness and softness of the crumb may be connected to moisture retention or the MD’s ability to prevent amylose retrogradation. It is significant to notice that the firmness of the control and combinations increased with storage time, with the exception of the sample containing 1.5% MD and 0.5% ZG. The lower crumb hardness found in samples containing 1% + 1% gum and MD may have been caused by the increased moisture content of these loaves, because the inverse link between hardness and moisture content has been widely documented. Because the BF1 and BF2 showed the maximum water absorption during dough mixing, yet the control and BF4 showed the least firm bread crumbs after 24 h, this characteristic cannot be entirely attributed to water absorption during dough formation. Therefore, the ability of the dough to maintain its moisture level during baking determines how hard the crumbs will be. This may help to explain why the BF5 mixture had the least firm crumbs after 96 h of storage. It is also important to highlight that the combination of MD and ZG outperformed the two components acting separately; more precisely, a combination consisting of more maltodextrins and less ZG. Additionally, after 24 h, a mixture with equal parts MD and ZG performed best, but after that, the mixture with three times as much MD reduced bread firmness the most. These results are consistent with observations in the literature on how hydrocolloids affect the hardness of bread [37]. According to literature reports, maltodextrins with a degree of polymerization (DP) between 4 and 66 decreases retrogradation, and hence lessens bread staling [21]. Additionally, given that DP 18 was employed in the study, it is clear how DPs in the range of 18 can function comparably to DPs at 66 in terms of bread firmness.

### 2.7. Bread Sensory Evaluation

**Figure 3:**
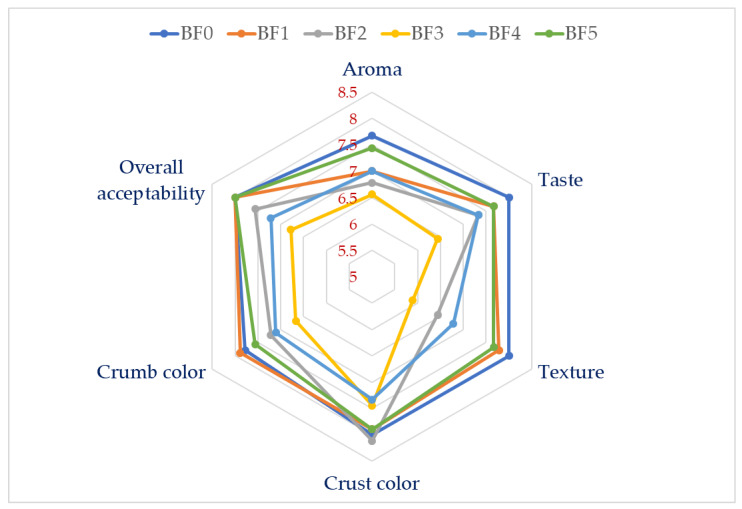
Effects of maltodextrin and ziziphus gums on the sensory evaluation score of bread samples. BF: control (100% wheat flour). BF1: 98% wheat flour + 2% maltodextrin. BF2: 98% wheat flour + 2% ziziphus gum. BF3: 98% wheat flour + 0.5% maltodextrin + 1.5% ziziphus gum. BF4: 98% wheat flour + 1% maltodextrin + 1% ziziphus gum. BF5: 98% wheat flour + 1.5% maltodextrin + 0.5% ziziphus gum.

The average results of the sensory assessment of bread prepared with the MD and ZG gums under investigation are displayed in Figure 3. In terms of aroma, there were no significant variations between the control and the mixes; however, samples BF2 and BF3 had lower scores, since they contained more ZG. MD and ZG both greatly impacted the taste negatively, although ZG did so more than MD. Samples containing ZG had the lowest texture scores, whereas samples with more MD had texture scores that were equivalent to the control. Although there was no discernible variation in the color of the crust and crumb between the control and the mixes, the sample containing ZG performed worse. Without any statistical significance, panelists generally agreed that loaves made from mixes were similarly acceptable, although samples containing ZG performed the worst when compared to MD and the control.

## 3. Materials and Methods

### 3.1. Materials

Maltodextrins (MD) with dextrose equivalent (DE 18) was purchased from Myprotein company (Myprotein, Gadbrook Park, CW9 7RA, Rudheath, Northwich, UK); bread wheat flour, yeast, salt, sugar, shortening and fat-free milk powder, were purchased from a local supermarket, Riyadh, Saudi Arabia. Wheat flour contained 13.6% protein, 11.1% moisture, 0.5% ash, 63.0% water absorption and 34 g wet gluten.

### 3.2. Methods

#### 3.2.1. Ziziphus Gum Extraction

The extraction of ziziphus gum (ZG) was done according to the method of Alamri, Mohamed, Hussain, Ibraheem, Qasem, Shamlan, Hakeem and Ababtain [15] with minor changes. Ziziphus fruits were collected from King Saud University farm in Riyadh. Fruits were carefully washed, destoned and steamed for 3 min to prevent enzymatic browning. The pulp was prepared by blending the fruits in a heavy-duty blender (BioloMix, Whirlpool corporation, Benton Harbor, MI, USA) (1:3 fruits to water ratio), filtered through a muslin cloth and centrifuged (Herolab GmbH Laborgeräte, Wiesloch, Germany) for 20 min at 3000× *g*. The supernatant was collected, neutralized (with NaOH), and freeze-dried. Freeze-dried gums were ground into powder using a coffee grinder, sieved using a 60-mesh sieve, and kept refrigerated in sealed jars.

#### 3.2.2. Preparation of Flour Mixes

Wheat flour, maltodextrins and ziziphus gum mixes were prepared by the replacement of the wheat flour with maltodextrins or ziziphus gum at different levels as listed below (Table 8).

#### 3.2.3. Solvent Retention Capacity (SRC)

Using AACC technique no. 56-11, the solvent retention capacity (SRC) of the flour mixes was assessed [38]. We used lactic acid (5% *v*/*v*), sodium bicarbonate (5% *v*/*v*), sugar (50% *v*/*v*), and double-distilled water as solvents. In 30 mL tubes, 1.0 g samples of flour or mixes were added to 25 mL of the prepared solvents, allowed to stand for 15 min, and then centrifuged at 1000× *g* for 20 min (Fisherbrand^TM^ Refrigerated Centrifuge GT2, Hamburg, Germany). The weight of the precipitated gels was determined after decanting the liquid, and the %SRC values for each solvent were calculated as follows:

$$\mathrm{SRC}\;(\%)=\frac{wet\;pellet\;\left(g\right)}{\left[flour\;weight\;\left(g\right)-1\right]}\;\times \;\frac{86}{\left[100-flour\;moisture\;\left(\%\right)\right]}\;\times \;100$$

#### 3.2.4. Pasting Behavior of Flour Mixes

Pasting qualities were measured using a Rapid Visco Analyzer (RVA, Newport Scientific, Sydney, Australia). The control (3.5 g at 14% moisture basis) or the mixtures were weighed directly into specific RVA canisters, and distilled water was then added to make a final weight of 28 g. The test was performed using only plain wheat flour as well as mixes. The slurry was heated to 50 °C for 30 s, followed by 4.40 min of 95 °C heating (at a rate of 10.23 °C/min), and then kept at 95 °C for 4 min. In 4 min, the sample was cooled to 50 °C, and it was then held there for 2 min [39]. The paddle was rotated at 960 rpm for the first 10 s before slowing to 160 for the remainder of the test. The profile of the tested samples includes the setback, final viscosity, and peak viscosity of the generated gel. The manufacturer’s Thermocline window software was used to process the data.

#### 3.2.5. Gel Texture

The RVA-made wheat flour gels were transferred to a 25 mL beaker and left there overnight at room temperature. Using a TA-TXT Texture Analyzer cylinder probe, the gels were compressed at a rate of 0.5 mm/s throughout the course of two penetration cycles for a 10 mm depth with a 12.7 mm-wide and 35 mm-long cylindrical probe (Vienna Court, Lammas Road, UK). The gel’s hardness, cohesiveness, adhesiveness, and gumminess were all measured and calculated in accordance with Sandhu and Singh [40].

#### 3.2.6. Dough Mixing Properties Using DoughLab

The Micro-DoughLab (Perten Instruments, Sidney, Australia) was used to determine the ideal water absorption capacity required to reach a peak of 500 FU within 2.0 min using a 4.00 ± 0.01 g sample at 14% moisture base after moisture correction. The samples were blended for 20 min at a speed of 63 rpm at a temperature of 30 °C. For each mixture, profiles with and without maltodextrins (MD) or ziziphus (ZG) (2% or 5%) were collected. It was determined how long the dough took to form and how stable it was. The dough stability is the difference in time between the top of the curve’s first intersection with the 500 FU line (arrival time) and its final departure from the 500 FU line (departure line). The difference in FU between the peak of the curve and five minutes later is known as the mixing tolerance index (MTI). The farinograph quality number (FQN) is the distance along the timeline from the start of water addition until the point where the center of the curve is 30 FU lower than at the development time. The degree of softening describes the difference between the consistency value of the curve center at the end of the developing time and the curve center 12 min after the developing time. The parameters were measure according to the method followed by Dangi, et al. [41]

#### 3.2.7. Dough Extensibility

Dough extensibility was determined using a texture analyzer (Stable Micro Systems, Godalming, UK). The extent of dough extensibility was assessed using the Al-Saleh and Brennan [42] method. After 2% sodium chloride was added to the flour or mix in place of the original amount, the dough was made in the Micro-DoughLab in accordance with the proper water absorption and development period (Table 3). The samples were made in accordance with the Kieffer extensibility rig’s specifications. The dough balls were then put on a dough clamp and left to rest at room temperature for 40 min. The sample plate was loaded with the dough strips and deposited onto the sample holder of the device. To assess the extensibility of dough, the Texture Analyzer (TA-XT plus, Stable Micro Systems, Godalming, Surrey, UK) was calibrated for a load cell of 50 kg. The Kieffer rig’s tensile test was performed to assess extensibility, with the following parameters employed: pre-test speed of 2.0 mm/s; test speed of 3.3 mm/s; post-test speed of 10.0 mm/s; distance of 75 mm; trigger force of auto-5 g; and data rate acquisition of 200 points per second.

#### 3.2.8. Bread Baking Procedures

The AACC method No. 10-09 was used to evaluate the baking performance of the mixes and the control flour [38]. According to the weight of the flour (100 g), the following dry ingredients were added: 3 g instant dry yeast, 6 g sugar, 0.02 g improver (α-amylase and vitamin C), 4 g fat-free instant milk powder, 1.5 g salt (NaCl), and 5 g shortening. The flour control was made of 100% wheat flour. The right amount of water was used to make the dough for the control and each blend. The method of straight dough was applied to make panned bread. Prior to and following punching, the dough was proofed for a total of 120 min. The dough was sheeted, molded, panned, and proofed (30 °C, 85% relative humidity) for a further 30 min prior to baking after the second proofing. All bread loaves were baked for 20 min at 220 °C in a hearth-style oven (National MFG. Co., Lincoln, NE 68508, USA). The baked loaves were cut into slices and stored for subsequent testing after being allowed to cool at room temperature.

#### 3.2.9. Bread Firmness after Storage

According to AACC method No. 74-09 [38], firmness was assessed after 24 and 96 h using two central loaf slices that were each 25 mm thick. Bread samples were maintained at room temperature. The TA-TXT texture analyzer (Stable Micro Systems, Surrey, UK), which has a 50 kg compression cell, was used with a cylindrical probe (20 mm) mounted on it. A bread sample (25 mm thick) was compressed by 25% strain, and the depth of the compression was 6.25 m. When the probe had returned to its initial location after spending 60 s at this distance in the slice, the percentage of springiness was computed. For tests on bread products, the pretest and test speeds were set to 1.0 mm/s. The firmness was determined by the compression force value (CFV), and the Stable Microsystem’s Exponent software was used to process the data.

#### 3.2.10. Crumb Color of Bread

Using a Chroma meter Minolta color grader with a D65 light source (Konica Minolta CR-40), color characteristics including the L* (lightness), a* (redness), and b* (yellowness) of bread and cake crumb samples were assessed [43].

#### 3.2.11. Sensory Evaluation of Breads

Panelists from the Department of Food Science and Nutrition at King Saud University were chosen from among staff and graduate students. The attributes of the bread samples such as aroma, taste, texture, and general acceptability were graded on a 9-point hedonic scale by the evaluators, with 9.0 denoting extremely good and 1.0 denoting extremely poor [44]. A skilled group of judges conducted a sensory analysis of the bread samples. The panel’s (10 members) members received training on how to distinguish between samples of control bread baked with various formulas. The sensory panel was made up of people who could tell which bread samples were similar and which ones were different. Due to the widespread consumption of bread in this society, all of the panelists were familiar with them. The 10 panelists were asked to evaluate the breads for attributes such as volume, symmetry of form, crust color, character of crust, evenness of bake, texture, crumb color, grain, taste and aroma, and overall acceptability.

#### 3.2.12. Statistical Analysis

All the measurements were done in triplicate. A one-way analysis of variance (ANOVA) was adopted to analyze the obtained data and estimate the significance (at *p* ≤ 0.05) of the effect of maltodextrin and ziziphus gum levels on the rheological, physio-chemical, quality, and sensory properties of pan bread. The PASW^®^ 18 program utilized the Duncan’s multiple range (DMR) test to compare means (SPSS Inc., Hong Kong, China).

## 4. Conclusions

By including MD and ZG, especially MD, the time required to mix the dough and water absorption was increased. The mixes’ dough stability increased, with the exception of samples that contained ZG. With the exception of the samples with 2% MD, which indicated a reduced dough tolerance in the middle of mixing time, the MTI of the mixes increased. ZG and MD considerably increased the extensibility of the dough. Due to the larger ZG content in the mix, bread firmness increased, but MD functioned better. Compared to the control and the other mixes, the combination high in MD (1.5% MD + 0.5% ZG) prevented a firmness increase after 96 h of storage. The mixes’ loaf volumes decreased compared to the control, but the presence of more MD kept it at a level that was closer to the control. The control loaf’s color was in the middle of the darkest (BF2, BF3, BF4) and lightest (BF1 and BF5) samples, indicating that ZG lessens the crumb’s lightness. The color of the crumb showed a mixed response to MD and ZG, with a darker crumb recorded for both MD and ZG mixes, but a lighter color for mixes with more MD. The panelists generally accepted the bread made from mixes in a way similar to the control. The MD or ZG utilized here had a significant impact on how hydrocolloids affected the bread’s sensory quality. The present research concludes that both types of hydrocolloids can be used in various combinations to produce quality bread without compromising the technological, functional and sensory attributes.

## Figures and Tables

**Table 1:** Pasting properties of wheat flour and mixes.

Mixes	PV (cP)	BD (cP)	FV (cP)	SB (cP)	PT (°C)
* BF	2694.00 ± 22.72 ^a^	983.67 ± 88.23 ^a^	3105.67 ± 42.10 ^a^	1395.33 ± 67.53 ^a^	70.48 ± 0.94 ^b^
BF1	2522.33 ± 11.55 ^c^	969.00 ± 15.72 ^a^	2929.00 ± 12.29 ^d^	1375.67 ± 17.79 ^a^	81.32 ± 8.98 ^a^
BF2	2609.67 ± 36.46 ^b^	1043.33 ± 17.62 ^a^	2990.33 ± 31.79 ^b^	1424.00 ± 21.17 ^a^	67.17 ± 0.46 ^b^
BF3	2576.67 ± 12.10 ^bc^	1013.33 ± 51.98 ^a^	2943.33 ± 17.01 ^cd^	1380.00 ± 50.41 ^a^	67.37 ± 0.54 ^b^
BF4	2574.00 ± 50.71 ^bc^	962.00 ± 78.58 ^a^	2975.33 ± 14.74 ^bc^	1363.33 ± 81.40 ^a^	68.27 ± 0.89 ^b^
BF5	2569.33 ± 24.58 ^bc^	1007.33 ± 26.01 ^a^	2964.67 ± 6.81 ^bcd^	1402.67 ± 8.39 ^a^	67.70 ± 0.05 ^b^

cP = centipoise. PT = Peak temperature. BD = Breakdown. FV = Final viscosity. SB = setback. * BF: control (100% wheat flour). BF1: 98% wheat flour + 2% maltodextrin. BF2: 98% wheat flour + 2% ziziphus gum. BF3: 98% wheat flour + 0.5% maltodextrin + 1.5% ziziphus gum. BF4: 98% wheat flour + 1% maltodextrin + 1% ziziphus gum. BF5: 98% wheat flour + 1.5% maltodextrin + 0.5% ziziphus gum. Means carrying different letters in a column are significantly (*p* ≤ 0.05) different from each other.

**Table 2:** Effects of maltodextrin and ziziphus gums on the properties of flour gels.

	Hardness (g)	Gumminess (g)	Chewiness (g)	Springiness (mm)	Cohesiveness	Adhesiveness (mJ)
* BF	85.00 ± 7.55 ^a^	45.51 ± 2.36 ^a^	516.43 ± 149.84 ^a^	11.27 ± 2.71 ^a^	0.54 ± 0.02 ^a^	0.73 ± 0.47 ^a^
BF1	77.33 ± 6.11 ^ab^	39.45 ± 3.42 ^b^	388.07 ± 35.67 ^b^	9.83 ± 0.06 ^a^	0.51 ± 0.01 ^ab^	0.93 ± 0.06 ^a^
BF2	70.33 ± 1.53 ^bc^	34.21 ± 1.33 ^c^	333.05 ± 16.02 ^b^	9.73 ± 0.12 ^a^	0.49 ± 0.03 ^b^	1.07 ± 0.05 ^a^
BF3	70.67 ± 2.08 ^bc^	34.41 ± 1.82 ^c^	337.51 ± 27.26 ^b^	9.80 ± 0.26 ^a^	0.49 ± 0.01 ^b^	0.97 ± 0.23 ^a^
BF4	73.67 ± 4.04 ^bc^	37.80 ± 1.76 ^bc^	378.03 ± 21.86 ^b^	10.00 ± 0.36 ^a^	0.51 ± 0.01 ^ab^	0.90 ± 0.30 ^a^
BF5	67.33 ± 6.11 ^c^	33.68 ± 3.52 ^c^	337.00 ± 38.27 ^b^	10.00 ± 0.10 ^a^	0.50 ± 0.02 ^b^	0.90 ± 0.20 ^a^

* BF: control (100% wheat flour). BF1: 98% wheat flour + 2% maltodextrin. BF2: 98% wheat flour + 2% ziziphus gum. BF3: 98% wheat flour + 0.5% maltodextrin + 1.5% ziziphus gum. BF4: 98% wheat flour + 1% maltodextrin + 1% ziziphus gum. BF5: 98% wheat flour + 1.5% maltodextrin + 0.5% ziziphus gum. Means carrying different letters in a column are significantly (*p* ≤ 0.05) different from each other.

**Table 3:** Effects of maltodextrin and ziziphus gums on the dough mixing properties of flour mixes.

	WA as Is (%)	DDT (min)	Stability (min)	Softening (FU)	MTI (FU)	FQN (mm)
* BF	56.20 ± 1.21 ^c^	1.70 ± 0.20 ^c^	5.93 ± 0.98 ^bc^	81.67 ± 15.27 ^bc^	35.00 ± 5.00 ^b^	63.43 ± 2.65 ^ab^
BF1	59.00 ± 0.00 ^a^	6.43 ± 0.65 ^a^	9.93 ± 0.32 ^a^	73.33 ± 2.88 ^c^	38.33 ± 5.77 ^b^	64.47 ± 2.30 ^a^
BF2	59.00 ± 0.00 ^a^	4.33 ± 0.37 ^ab^	5.80 ± 0.34 ^c^	103.33 ± 17.55 ^ab^	60.00 ± 13.22 ^ab^	55.07 ± 4.86 ^c^
BF3	58.00 ± 0.00 ^b^	4.77 ± 0.25 ^ab^	6.20 ± 0.34 ^bc^	111.67 ± 7.63 ^a^	66.67 ± 5.77 ^a^	52.43 ± 3.01 ^c^
BF4	57.87 ± 0.11 ^b^	3.93 ± 1.74 ^abc^	6.50 ± 0.40 ^bc^	108.33 ± 12.58 ^a^	50.00 ± 21.79 ^ab^	56.03 ± 4.57 ^bc^
BF5	57.20 ± 0.00 ^b^	3.27 ± 2.63 ^bc^	7.03 ± 0.87 ^b^	98.33 ± 10.40 ^ab^	35.00 ± 18.02 ^b^	60.03 ± 6.04 ^abc^

* BF: control (100% wheat flour). BF1: 98% wheat flour + 2% maltodextrin. BF2: 98% wheat flour + 2% ziziphus gum. BF3: 98% wheat flour + 0.5% maltodextrin + 1.5% ziziphus gum. BF4: 98% wheat flour + 1% maltodextrin + 1% ziziphus gum. BF5: 98% wheat flour + 1.5% maltodextrin + 0.5% ziziphus gum. WA = Water absorption. DDT = Dough development time. FU = Farino units. MTI = Mixing tolerance index. Farinograph Quality Number. Means carrying different letters in a column are significantly (*p* ≤ 0.05) different from each other.

**Table 4:** Effects of maltodextrin and ziziphus gums on the dough extensibility of flour mixes.

	Resistance to Extension (g)	Extensibility (mm)
* BF	78.03 ± 2.43 ^a^	18.78 ± 1.66 ^c^
BF1	80.29 ± 1.48 ^a^	40.69 ± 4.80 ^ab^
BF2	44.58 ± 1.29 ^c^	29.84 ± 5.39 ^b^
BF3	59.73 ± 3.89 ^b^	39.87 ± 7.54 ^ab^
BF4	77.88 ± 1.64 ^a^	42.33 ± 6.89 ^a^
BF5	56.50 ± 2.28 ^b^	32.74 ± 6.85 ^ab^

* BF: control (100% wheat flour). BF1: 98% wheat flour + 2% maltodextrin. BF2: 98% wheat flour + 2% ziziphus gum. BF3: 98% wheat flour + 0.5% maltodextrin + 1.5% ziziphus gum. BF4: 98% wheat flour + 1% maltodextrin + 1% ziziphus gum. BF5: 98% wheat flour + 1.5% maltodextrin + 0.5% ziziphus gum. Means carrying different letters in a column are significantly (*p* ≤ 0.05) different from each other.

**Table 5:** Effects of maltodextrin and ziziphus gums on the solvent retention capacity properties of flour mixes.

	WRC	SuSRC	SCRC	LARC	GPI
* BF	76.07 ± 7.97 ^b^	131.51 ± 27.54 ^a^	97.67 ± 5.12 ^ab^	137.96 ± 19.08 ^b^	0.60 ± 0.06 ^b^
BF1	88.00 ± 0.97 ^ab^	84.45 ± 9.54 ^b^	78.49 ± 8.60 ^b^	131.19 ± 2.44 ^b^	0.81 ± 0.03 ^a^
BF2	87.03 ± 0.97 ^b^	128.29 ± 8.78 ^a^	114.75 ± 20.84 ^a^	130.55 ± 0.00 ^b^	0.54 ± 0.03 ^b^
BF3	83.16 ± 0.97 ^a^	110.88 ± 17.94 ^ab^	98.63 ± 4.22 ^ab^	160.52 ± 12.79 ^ab^	0.77 ± 0.09 ^a^
BF4	92.83 ± 5.39 ^a^	127.32 ± 15.72 ^a^	97.18 ± 13.06 ^ab^	182.76 ± 8.60 ^a^	0.81 ± 0.06 ^a^
BF5	82.20 ± 0.97 ^a^	97.34 ± 24.03 ^ab^	101.21 ± 2.79 ^a^	154.72 ± 30.29 ^ab^	0.78 ± 0.13 ^a^

* BF: control (100% wheat flour). BF1: 98% wheat flour + 2% maltodextrin. BF2: 98% wheat flour + 2% ziziphus gum. BF3: 98% wheat flour + 0.5% maltodextrin + 1.5% ziziphus gum. BF4: 98% wheat flour + 1% maltodextrin + 1% ziziphus gum. BF5: 98% wheat flour + 1.5% maltodextrin + 0.5% ziziphus gum. WRC = Water retention capacity. SuSRC = Sucrose retention capacity. SCRC = Sodium carbonate retention capacity. LARC = Lactic acid retention capacity; GPI = Gluten performance index. Means carrying different letters in a column are significantly (*p* ≤ 0.05) different from each other.

**Table 6:** Effects of maltodextrin and ziziphus gums on the volume and weight of bread samples.

	Loaf Volume (cm^3^)	Loaf Weight (g)	Specific Volume (cm^3^/g)
* BF	1371.37 ± 2.19 ^a^	471.80 ± 2.50 ^c^	2.91 ± 0.02 ^a^
BF1	1231.67 ± 1.89 ^c^	481.75 ± 0.45 ^a^	2.56 ± 0.01 ^d^
BF2	1051.15 ± 3.56 ^d^	468.35 ± 1.65 ^d^	2.25 ± 0.01 ^e^
BF3	901.17 ± 2.29 ^e^	461.25 ± 2.05 ^e^	1.95 ± 0.02 ^f^
BF4	1291.88 ± 5.23 ^b^	479.20 ± 0.30 ^ab^	2.70 ± 0.01 ^c^
BF5	1291.66 ± 11.81 ^b^	476.05 ± 2.65 ^b^	2.71 ± 0.01 ^b^

* BF: control (100% wheat flour). BF1: 98% wheat flour + 2% maltodextrin. BF2: 98% wheat flour + 2% ziziphus gum. BF3: 98% wheat flour + 0.5% maltodextrin + 1.5% ziziphus gum. BF4: 98% wheat flour + 1% maltodextrin + 1% ziziphus gum. BF5: 98% wheat flour + 1.5% maltodextrin + 0.5% ziziphus gum. Means carrying different letters in a column are significantly (*p* ≤ 0.05) different from each other.

**Table 7:** Effects of maltodextrin and ziziphus gums on the crumb color parameters of bread samples.

	L*	a*	b*
* BF	75.37 ± 0.98 ^ab^	−2.85 ± 0.18 ^c^	14.48 ± 0.36 ^b^
BF1	77.00 ± 1.35 ^a^	−2.82 ± 0.10 ^c^	15.63 ± 0.34 ^ab^
BF2	74.70 ± 0.56 ^b^	−2.02 ± 0.05 ^ab^	16.73 ± 0.31 ^a^
BF3	74.59 ± 0.28 ^b^	−1.91 ± 0.22 ^a^	16.73 ± 0.60 ^a^
BF4	74.58 ± 0.67 ^b^	−2.28 ± 0.06 ^b^	16.07 ± 1.13 ^a^
BF5	76.86 ± 1.00 ^a^	−2.64 ± 0.22 ^c^	15.35 ± 1.12 ^ab^

* BF: control (100% wheat flour). BF1: 98% wheat flour + 2% maltodextrin. BF2: 98% wheat flour + 2% ziziphus gum. BF3: 98% wheat flour + 0.5% maltodextrin + 1.5% ziziphus gum. BF4: 98% wheat flour + 1% maltodextrin + 1% ziziphus gum. BF5: 98% wheat flour + 1.5% maltodextrin + 0.5% ziziphus gum. L* = lightness; a* = green/red; b* = blue/yellow. Means carrying different letters in a column are significantly (*p* ≤ 0.05) different from each other.

**Table 8:** Composition of flour mixes.

Mixes	Wheat Flour (%)	Maltodextrins (%)	Ziziphus Gum (%)
BF0	100	-	-
BF1	98	2	-
BF2	98	-	2
BF3	98	0.5	1.50
BF4	98	1.00	1.00
BF5	98	1.50	0.50

BF0 = 100% wheat flour (control). BF1 = 98% BF + 2% maltodextrins. BF2 = 98% BF + 2% ziziphus gum. BF3 = 98% BF+ 0.5% maltodextrins + 1.5% ziziphus gum. BF4 = 98% BF + 1% maltodextrins + 1% ziziphus gum. BF5 = 98% BF + 1.5% maltodextrins + 0.5% ziziphus gum.

## Data Availability

Not applicable.

## References

[1] Collar C., Andreu P., Martınez J., Armero E. (1999). Optimization of hydrocolloid addition to improve wheat bread dough functionality: A response surface methodology study. Food Hydrocoll..

[2] Lazaridou A., Duta D., Papageorgiou M., Belc N., Biliaderis C.G. (2007). Effects of hydrocolloids on dough rheology and bread quality parameters in gluten-free formulations. J. Food Eng..

[3] Linlaud N., Puppo M., Ferrero C. (2009). Effect of hydrocolloids on water absorption of wheat flour and farinograph and textural characteristics of dough. Cereal Chem..

[4] Das L., Raychaudhuri U., Chakraborty R. (2015). Effects of hydrocolloids as texture improver in coriander bread. J. Food Sci. Technol..

[5] Bárcenas M.E., Rosell C.M. (2007). Different approaches for increasing the shelf life of partially baked bread: Low temperatures and hydrocolloid addition. Food Chem..

[6] Davidou S., Le Meste M., Debever E., Bekaert D. (1996). A contribution to the study of staling of white bread: Effect of water and hydrocolloid. Food Hydrocoll..

[7] Rosell C.M., Rojas J.A., De Barber C.B. (2001). Influence of hydrocolloids on dough rheology and bread quality. Food Hydrocoll..

[8] Shittu T.A., Aminu R.A., Abulude E.O. (2009). Functional effects of xanthan gum on composite cassava-wheat dough and bread. Food Hydrocoll..

[9] Rojas J.A., Rosell C.M., De Barber C.B. (1999). Pasting properties of different wheat flour-hydrocolloid systems. Food Hydrocoll..

[10] Delfanian M., Esmaeilzadeh Kenari R., Sahari M.A. (2016). Utilization of Jujube fruit (*Ziziphus mauritiana* Lam.) extracts as natural antioxidants in stability of frying oil. Int. J. Food Prop..

[11] Obeed R., Harhash M., Abdel-Mawgood A. (2008). Fruit properties and genetic diversity of five ber (*Ziziphus mauritiana* Lamk.) cultivars. Pak. J. Biol. Sci..

[12] Muchuweti M., Zenda G., Ndhlala A.R., Kasiyamhuru A. (2005). Sugars, organic acid and phenolic compounds of *Ziziphus mauritiana* fruit. Eur. Food Res. Technol..

[13] Ray P., Chatterjee S., Saha P. (2021). Screening of polysaccharides from fruit pulp of *Ziziphus mauritiana* L. and Artocarpus heterophyllus L. as natural mucoadhesives. Future J. Pharm. Sci..

[14] Lee H.B., Kim S.Y. (1988). Studies on the changes of chemical components of dried jujube (*Zizyphus jujuba* Miller) during storage. Korean J. Agric. Sci..

[15] Alamri M.S., Mohamed A.A., Hussain S., Ibraheem M.A., Qasem A.A.A., Shamlan G., Hakeem M.J., Ababtain I.A. (2022). Functionality of Cordia and Ziziphus Gums with Respect to the Dough Properties and Baking Performance of Stored Pan Bread and Sponge Cakes. Foods.

[16] Mohamed A., Hussain S., Alamri M.S., Ibraheem M.A., Qasem A.A.A., Ababtain I.A. (2022). Physicochemical Properties of Starch Binary Mixtures with Cordia and Ziziphus Gums. Processes.

[17] Wang Y.J., Wang L. (2000). Structures and properties of commercial maltodextrins from corn, potato, and rice starches. Starch-Stärke.

[18] Wang Y.-J., Jane J. (1994). Correlation between glass transition temperature and starch retrogradation in the presence of sugars and maltodextrins. Cereal Chem..

[19] Pycia K., Gryszkin A., Berski W., Juszczak L. (2018). The influence of chemically modified potato maltodextrins on stability and rheological properties of model oil-in-water emulsions. Polymers.

[20] Smits A., Kruiskamp P., Van Soest J., Vliegenthart J. (2003). The influence of various small plasticisers and malto-oligosaccharides on the retrogradation of (partly) gelatinised starch. Carbohydr. Polym..

[21] Defloor I., Delcour J.A. (1999). Impact of maltodextrins and antistaling enzymes on the differential scanning calorimetry staling endotherm of baked bread doughs. J. Agric. Food Chem..

[22] Palacios H.R., Schwarz P.B., D’Appolonia B.L. (2004). Effect of α-amylases from different sources on the retrogradation and recrystallization of concentrated wheat starch gels: Relationship to bread staling. J. Agric. Food Chem..

[23] Vedantam S.K., Sagili J.L., Dikkala P.K., Sridhar K. (2021). Functional, thermal, pasting, and rheological properties of gluten-free maize composite flour: Effect of moth bean flour and hydrocolloid addition. J. Food Process. Preserv..

[24] Alamri M., Mohamed A., Hussain S., Xu J. (2012). Effect of okra extract on properties of wheat, corn and rice starches. J. Food Agric. Environ..

[25] Sudhakar V., Singhal R.S., Kulkarni P.R. (1995). Studies on starch-hydrocolloid interactions: Effect of salts. Food Chem..

[26] Alloncle M., Lefebvre J., Llamas G., Doublier J. (1989). A rheological characterization of cereal starch-galactomannan mixtures. Cereal Chem..

[27] Alam F., Siddiqui A., Lutfi Z., Hasnain A. (2009). Effect of different hydrocolloids on gelatinization behaviour of hard wheat flour. Trakia J. Sci..

[28] Katsuta K., Nishimura A., Miura M. (1992). Effects of saccharides on stabilities of rice starch gels. 2. Oligosaccharides. Food Hydrocoll..

[29] Luo D., Li Y., Xu B., Ren G., Li P., Li X., Han S., Liu J. (2017). Effects of inulin with different degree of polymerization on gelatinization and retrogradation of wheat starch. Food Chem..

[30] Yildiz Ö., Yurt B., Baştürk A., Toker Ö.S., Yilmaz M.T., Karaman S., Dağlıoğlu O. (2013). Pasting properties, texture profile and stress–relaxation behavior of wheat starch/dietary fiber systems. Food Res. Int..

[31] Simsek S. (2009). Application of xanthan gum for reducing syruping in refrigerated doughs. Food Hydrocoll..

[32] Teng Y., Liu C., Bai J., Liang J. (2015). Mixing, tensile and pasting properties of wheat flour mixed with raw and enzyme treated rice bran. J. Food Sci. Technol..

[33] Gaines C. (2000). Collaborative study of methods for solvent retention capacity profiles (AACC Method 56-11). Cereal Foods World.

[34] Guttieri M.J., Becker C., Souza E.J. (2004). Application of wheat meal solvent retention capacity tests within soft wheat breeding populations. Cereal Chem..

[35] Maleki G., MilaNi J.M. (2013). Effect of guar gum, xanthan gum, CMC and HPMC on dough rhealogy and physical properties of Barbari bread. Food Sci. Technol. Res..

[36] Bell D.A. (1990). Methylcellulose as a structure enhancer in bread baking. Cereal Foods World.

[37] Filipčev B., Lević L., Bodroža-Solarov M., Mišljenović N., Koprivica G. (2010). Quality characteristics and antioxidant properties of breads supplemented with sugar beet molasses-based ingredients. Int. J. Food Prop..

[38] AACC (2000). Approved Methods of the American Association of Cereal Chemists.

[39] Sharma P., Gujral H.S. (2014). Cookie making behavior of wheat–barley flour blends and effects on antioxidant properties. LWT-Food Sci. Technol..

[40] Sandhu K.S., Singh N. (2007). Some properties of corn starches II: Physicochemical, gelatinization, retrogradation, pasting and gel textural properties. Food Chem..

[41] Dangi P., Chaudhary N., Khatkar B. (2019). Rheological and microstructural characteristics of low molecular weight glutenin subunits of commercial wheats. Food Chem..

[42] Al-Saleh A., Brennan C.S. (2012). Bread wheat quality: Some physical, chemical and rheological characteristics of Syrian and English bread wheat samples. Foods.

[43] Alamri M.S. (2014). Okra-gum fortified bread: Formulation and quality. J. Food Sci. Technol..

[44] Hussain S., Alamri M.S., Mohamed A.A., Ibraheem M.A., Qasem A.A.A., Shamlan G., Ababtain I.A. (2022). Exploring the Role of Acacia (*Acacia seyal*) and Cactus (*Opuntia ficus-indica*) Gums on the Dough Performance and Quality Attributes of Breads and Cakes. Foods.

